# Supplementary material for: Generative complex networks within a dynamic memristor with intrinsic variability
Source: Nat Commun. 2023 Oct 2;14:6134. doi: 10.1038/s41467-023-41921-3 (PMC10545788; doi:10.1038/s41467-023-41921-3)
Supplement: Supplementary file 1 — Supplementary Information [file 41467_2023_41921_MOESM1_ESM.pdf]

Supplementary information

Generative complex networks within a dynamic memristor with intrinsic variability

Yunpeng Guo[1+], Wenrui Duan[2+*], Xue Liu[3,1+*], Xinxin Wang[1], Lidan Wang[4], Shukai Duan[4], Cheng Ma[1*], Huanglong Li[1,5*]

[1]Department of Precision Instrument, Center for Brain Inspired Computing Research, Tsinghua University; Beijing, 100084, China

[2]School of Instrument Science and Opto Electronics Engineering, Laboratory of Intelligent Microsystems, Beijing Information Science & Technology University, Beijing, 100101, China

[3]School of Integrated Circuits, Tsinghua University, Beijing, 100084, China

[4]School of Artificial Intelligence, Southwest University, Chongqing, 400715, China

[5]Chinese Institute for Brain Research; Beijing, 102206, China

*Corresponding authors. Emails: li_huanglong@mail.tsinghua.edu.cn, duanwr10@buaa.edu.cn, liuqingxue@mail.tsinghua.edu.cn, macheng@tsinghua.edu.cn

[+]equal contribution

Supplementary Text

Comprehensive analyses of the characteristics of the PBAONC complex networks

The PBAONC mechanism can give rise to a range of complex networks of different numbers of edges. Since our main focus is complex networks for information processing, we consider two relevant measures: one is the clustering coefficient (C^*), a measure of information segregation (i.e., the degree to which a network is organized into local specialized regions), the other is the characteristic path length (L^*), a measure of information integration (i.e., the ability to rapidly combine pieces of specialized information from distributed regions). The contour plots of the landscapes of C^* and L^* as functions of D_{min} and D_{max} for networks (N=100) generated by the PBAONC mechanism are shown in Supplementary Fig. S5a,b with selected isolines of E (the expectation of the number of Edges) provided. It is seen that these isolines are almost though not strictly perpendicular to the right diagonal $D_{max}=D_{min}$ along which networks are parameterized to form regular connections. The upper right end of the diagonal corresponds to the FC network that has routinely been implemented with memristive virtual nodes for RC[1-4]. The gradients of C^* and L^* in the right diagonal direction (E varies monotonically) can be clearly observed. Specifically, sparsely connected networks (bottom left region) have long L^* and small C^*, whereas it is opposite for densely connected networks (upper right region). Many complex systems, such as the brain network, social network and the Internet, have evolved towards a most economical trade-off between minimizing wiring cost and maximizing efficiency[5, 6], characterized by small L^* and large C^*[7]. Supplementary Fig. S5c,d shows the L^* and C^* characteristics of the S-W SW network. This ubiquitous topological feature is famously known as the small-worldness. This also accounts for the optimal balance of functional segregation and integration in the brain network[8]. We also use scatter plots to visualize more clearly the evolution of C^* and L^* along given isolines of E. As shown in Supplementary Fig. S6**,** in the case of E=1200 (sparse connectivity), L^* decreases with increasing difference between D_{max} and D_{min} along the isoline (D_{max} and D_{min} vary monotonically but oppositely). As the connectivity becomes denser (E=3000), the changes of C^* and L^* along the isoline of E become increasingly flattened out. With further increase in the connection density (E=4800), the changes of C^* and L^* along the isoline of E, though still insignificant, show reversed trends, respectively, compared to those in sparsely connected network (E=1200).

Small-worldness of the PBAONC complex networks

To quantify the small-worldness of networks parameterized over the entire (D_{min}, D_{max}) space, we adopt metric S^* proposed by Humphries and Gurney[9] which is based on measuring the trade-off between high local clustering and short path length. As shown in Supplementary Fig. S7, the gradient of S^* is also most significant in the right diagonal direction. In agreement with that[9], when the number of nodes is fixed, S^* decays with increasing number of edges. Along an isoline of E, there is a unique point where S^* is maximized. For sparse connections (E=600, 1200), this point locates at the maximum $\{D_{max}\text{-}D_{min}\}$ end of the isoline; while for denser connections (E=1800, 2400, 3000, 3600, 4200), it is midway between the two ends.

Property comparison between the PBAONC complex network model and several canonical complex network models

In addition to C^*, L^* and S^*, we also quantify the properties of our complex network by other indicators and compare them with those of the other canonical complex networks, including the Watts–Strogatz (W-S) small-world (SW) network[7], Erdős–Rényi (E-R) random network[10] and Barabási–Albert (B-A) scale-free network[11], under the condition of same number of nodes (100) and same E, as shown in Supplementary Fig. S8. As a common reference, the FC network generated by our PBAONC mechanism is also included in each comparison group. It is seen that the FC network trivially has the smallest L^* and largest C^*. Its degree of small-worldness is low and comparable to that of the E-R random networks and B-A scale-free networks of varying E, from 600 to 4800. Because the FC network is itself a clique, i.e., a network that has connections between any two nodes within it, it has the largest 'maximum clique size'. Next to it, the PBAONC networks of different E in each comparison group always have the second largest 'maximum clique size'. This can be understood as due to the specific wiring rule in generating the PBAONC network, that is, any one node is connected to all its proximal neighbor nodes. For E=600, our PBAONC network has the largest C^*, and correspondingly, the largest 'local efficiency' (an indication of how effectively information is integrated between the immediate neighbors of individual nodes) among all complex networks. Its L^*, and correspondingly, 'radius', however, are still larger than those of the W-S SW network (the other two complex networks are known to have small L^*), therefore leading to the second highest degree of small-worldness next to the W-S SW network. As E increases, the differences in C^*, L^* and S^* between our PBAONC network and the W-S SW network become smaller, and the characteristics of all complex networks tend to be increasingly similar to that of the FC network, as expected. Nevertheless, the former two complex networks still have the highest degree of small-worldness. Measuring the efficiency of distant information transfer, the 'global efficiency' of our PBAONC network also improves as E increases. The treewidth measures the similarity between a graph and a tree. The PBAONC network has significantly smaller treewidth than any other networks and therefore it is the most tree-like network, suggesting that it is more likely to exhibit an intermediate-scale structure composed of small dense parts, representing clusters, that are sparsely interconnected. Again, this is a reflection of its specific wiring behavior that any one node is connected to all its proximal neighbor nodes (contributes to cluster formation) but forms no connection with any of its distal neighbors (contributes to the formation of sparse interconnections among clusters).

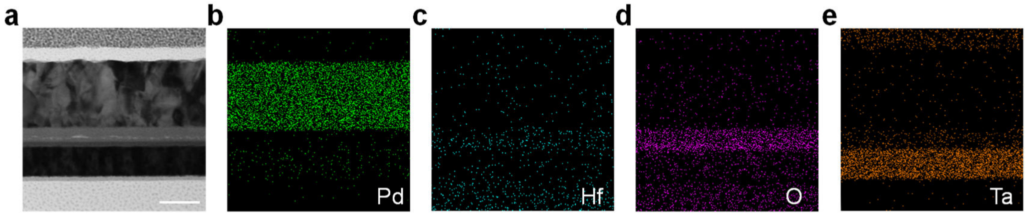

Fig. S1. Distribution of elements in the device. a Cross-sectional TEM image of the fabricated dynamic memristor (scale bar: 30 nm). EDS elemental mapping images of the device cross-section area where Pd is denoted in green (**b**), Hf in blue(**c**), O in purple (**d**), and Ta in orange (**e**).

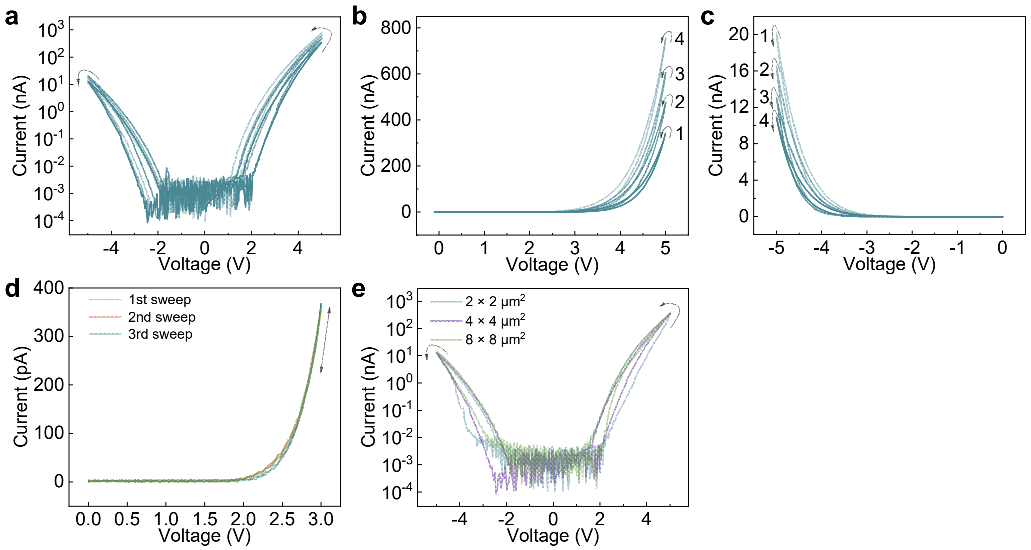

Fig. S2. Current–voltage (I–V) characteristics of the device. a Hysteretic I-V curves of a 4×4 μm^2 device obtained under four consecutive cyclic voltage sweeps between -5 V and + 5 V. **b** I-V curves obtained under four consecutive cyclic voltage sweeps between 0 V and + 5 V. The conductance of the device increases continuously as the number of sweeping cycles increases. **c** I-V curves obtained under four consecutive cyclic voltage sweeps between -5V and 0 V. The conductance of the device decreases continuously as the number of sweeping cycles increases. The I-V characteristics shown in Figs. S1a-c are not contradictory to the volatile switching characteristics obtained under pulse measurements (Fig. 1a in the manuscript) because the pulse stimulus is much more brief and therefore nonvolatile memristive changes may not be induced. **d** Single-valued (non-hysteretic) I-V curves obtained under three consecutive cyclic voltage sweeps between -3 V and +3 V. The absence of hysteresis in these I-V curves indicates that nonvolatile memristive changes do not take place in the device, which justifies the use of voltage pulses with the amplitudes of 3 V as the read voltage. **e** Comparison of the I-V curves of the devices with different areas (2×2 μm^2, 4×4 μm^2, 8×8 μm^2).

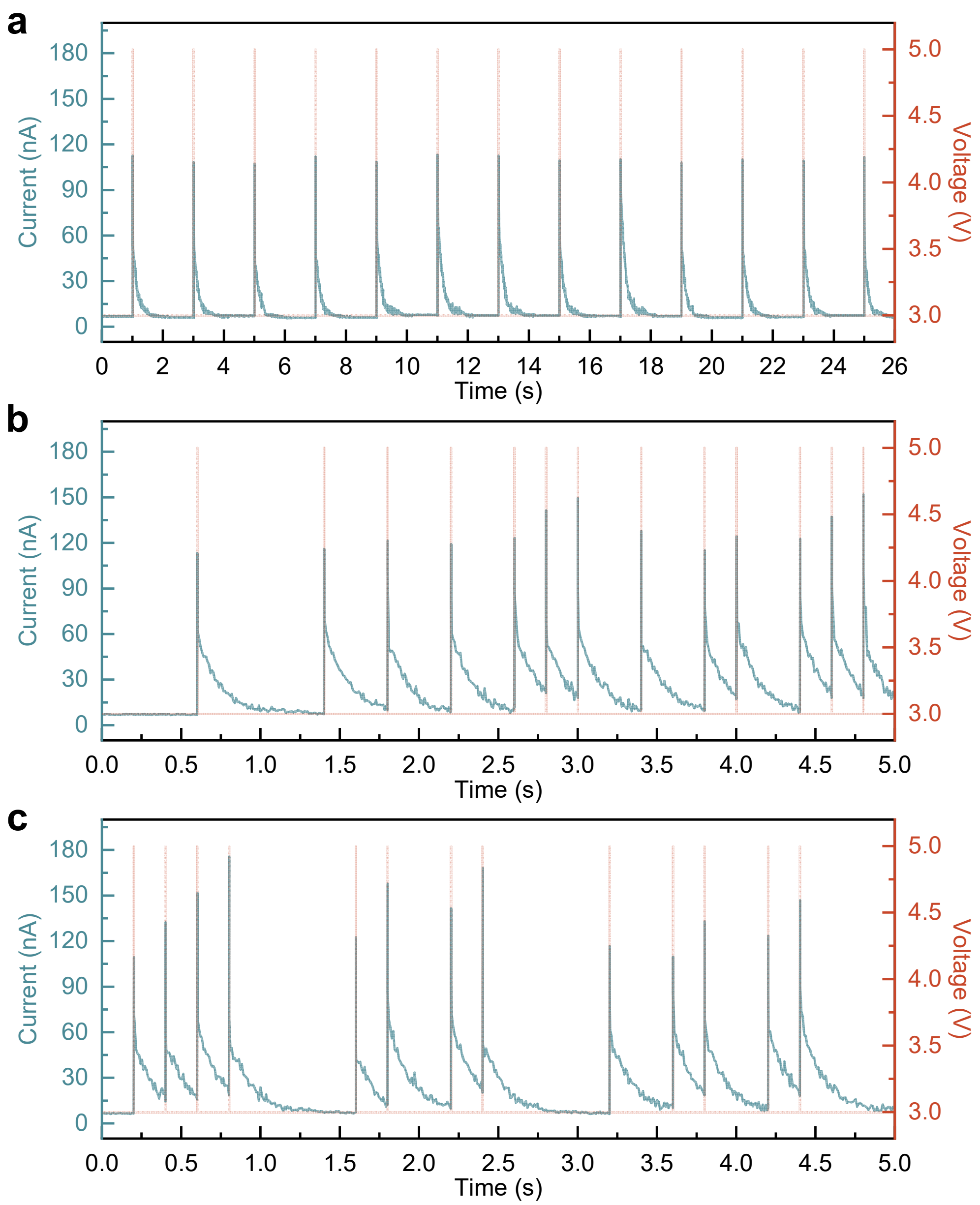

Fig. S3. The effect of the time-division multiplexing time step on the coupling among the virtual nodes. a Virtual nodes with a time interval of 2 s that is larger than τ_{max} are independent from each other, as manifested in the (almost) invariant current responses to each voltage pulse. **b, c** Virtual nodes with a time interval of 200 ms that is smaller than τ_{min} are dynamically coupled to their respective temporally adjacent nodes, as manifested in the different current evolution trajectories in response to different voltage pulse sequences.

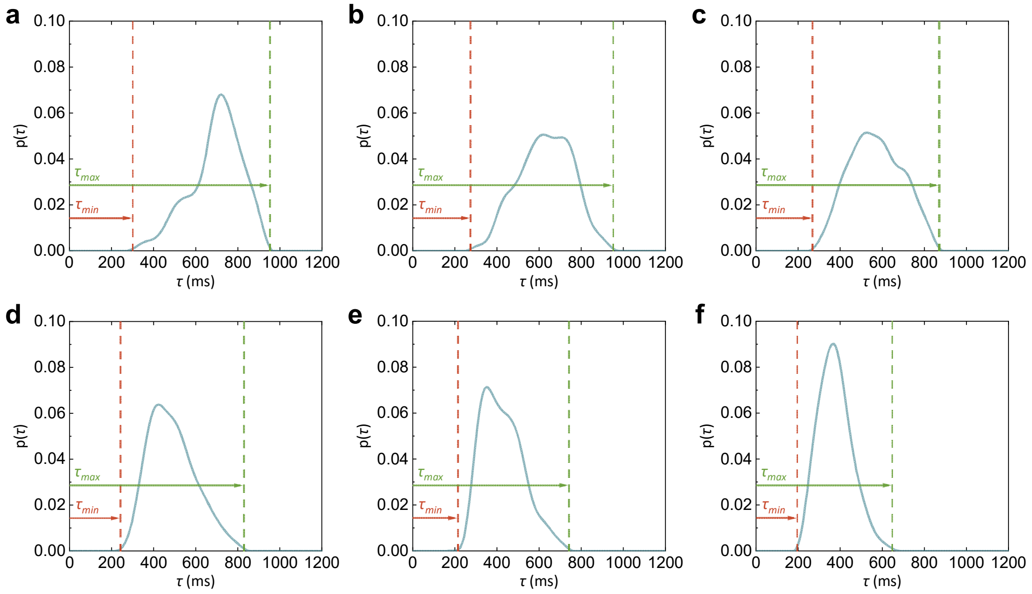

Fig. S4. Probability distribution of the current decay time *τ*. a-f Probability distributions of the decay time of I_+ (under 3-V read voltage) obtained under 1000 independent single-pulse measurements with the amplitudes of the stimulating pulses being 4.8 V (**a**), 4.6 V (**b**), 4.4 V (**c**), 4.2 V (**d**), 4.0 V (**e**) and 3.8 V (**f**), respectively.

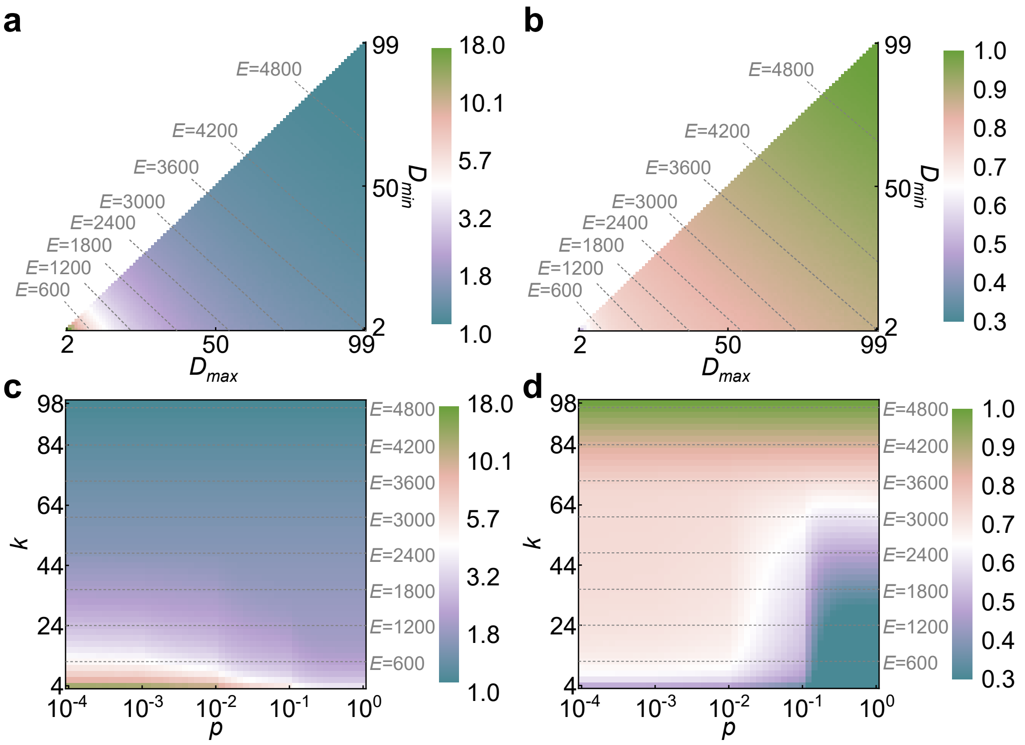

Fig. S5. Characteristic path lengths (L*s) and clustering coefficients (C*s) of the PBAONC complex networks and W-S SW networks with 100 nodes. a, b Contour plots of $L*$ (**a**) and $C*$ (**b**) as functions of D_{max} and D_{min} for the PBAONC network model with 100 nodes. The grey dash lines are the isolines of E (the expectation of the number of Edges). **c, d** Contour plots of $L*$ (**c**) and $C*$ (**d**) as functions of the random rewiring probability p and the number of connected nearest neighbors k in the baseline regular network. The grey dash lines are the isolines of E.

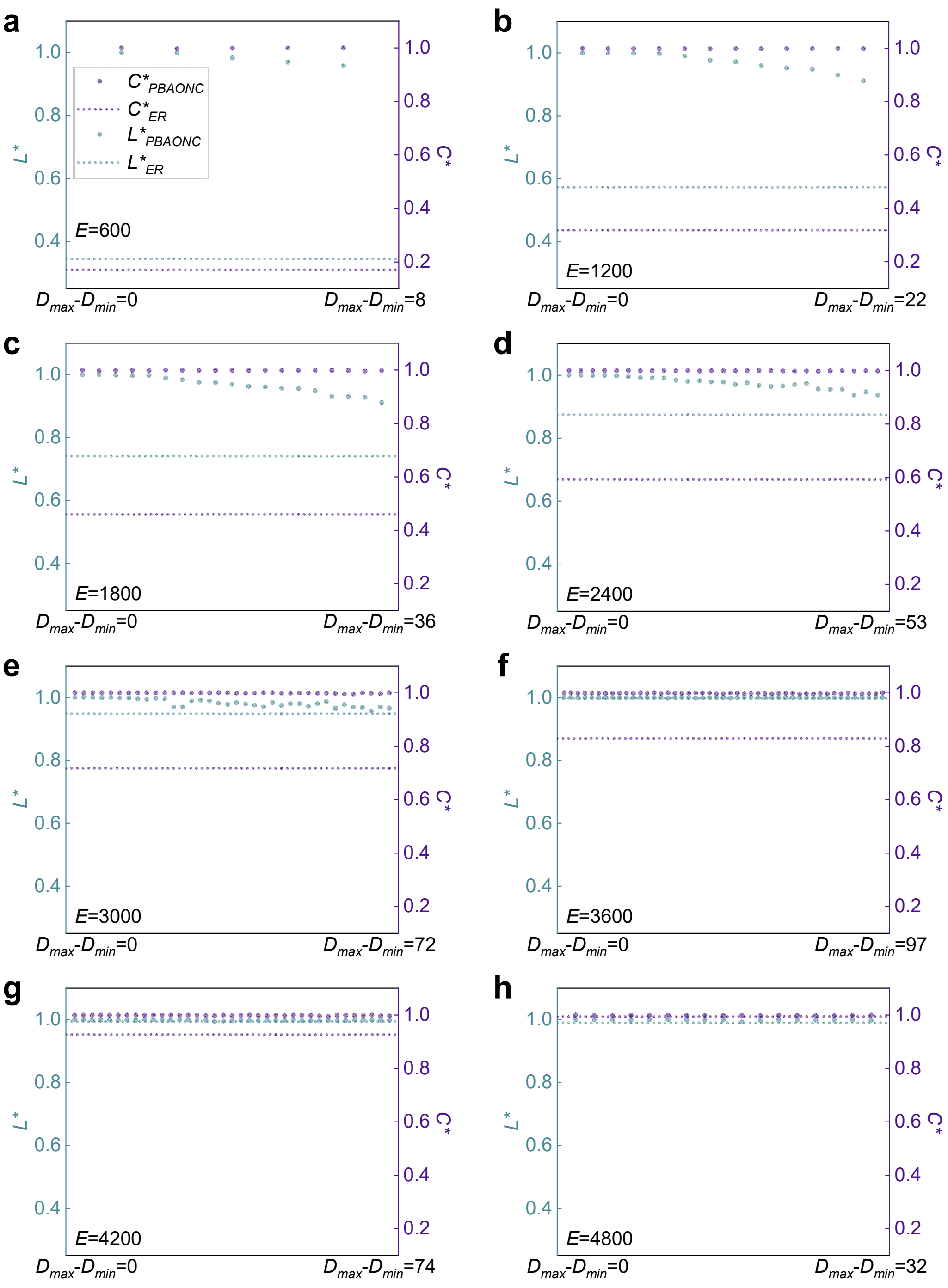

Fig. S6. Characteristic path lengths (*Ls) and clustering coefficients (*C**s) of the PBAONC networks. a-h** *L**s and *C**s of PBAONC network model and the E-R random network model along the isolines of *E*=600 (**a**), *E*=1200 (**b**), *E*=1800 (**c**), *E*=2400 (**d**), *E*=3000 (**e**), *E*=3600 (**f**), *E*=4200 (**g**) and *E*=4800 (**h**).

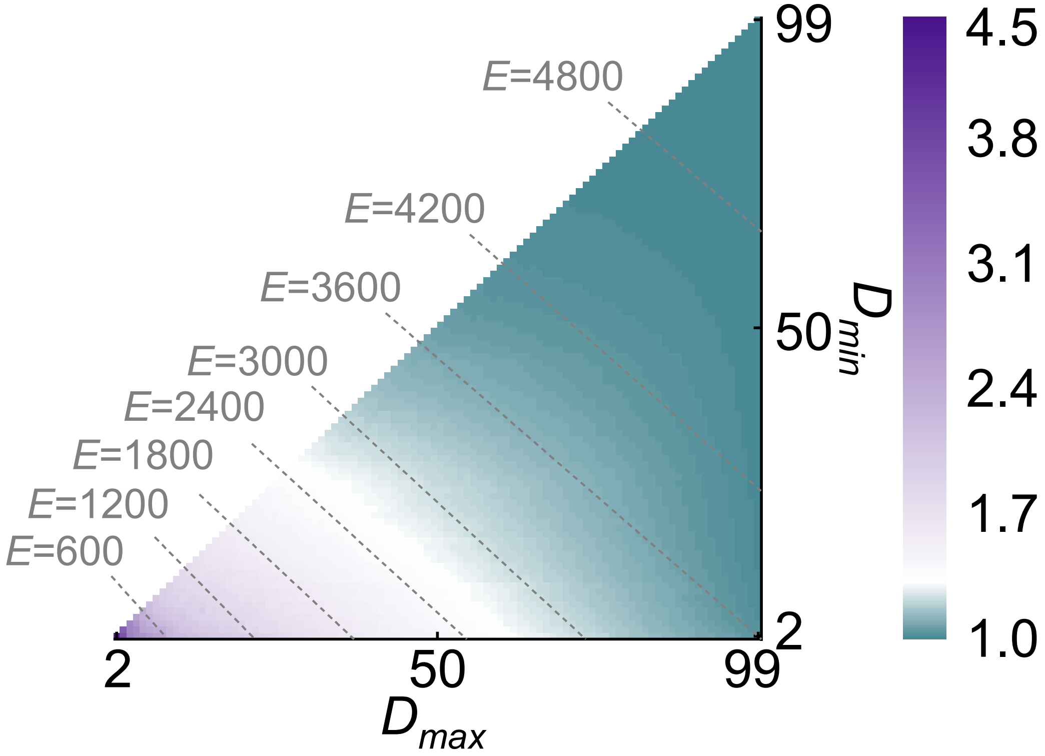

Fig. S7. Contour plots of *S as functions of the PBAONC network models with 100 nodes.**

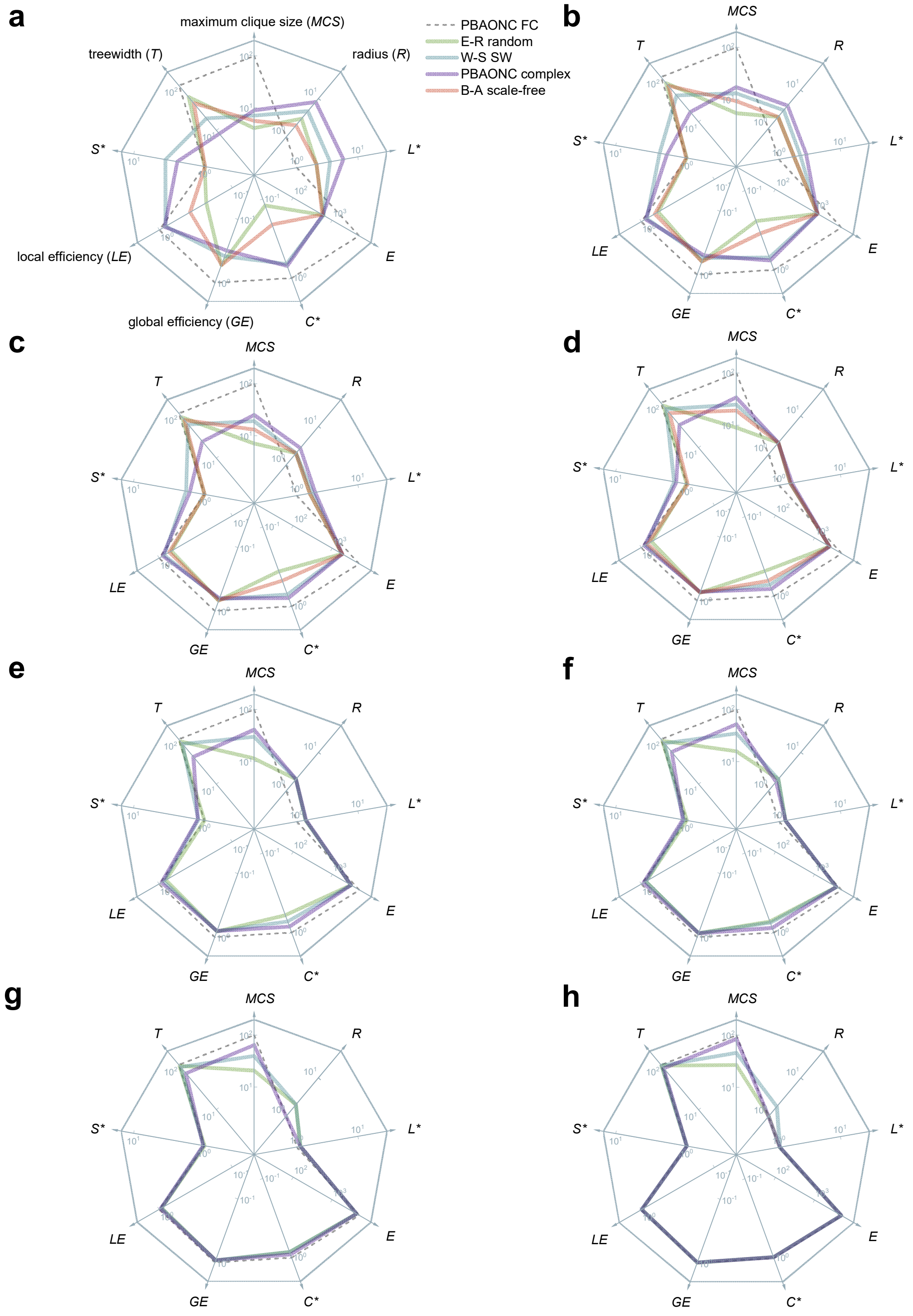

Fig. S8. Property comparison between the PBAONC complex network model and several canonical complex network models with different connection densities. a-h Radar charts of nine network topology indicators for the PBAONC FC networks, the E-R random networks, the W-S SW networks, the PBAONC complex networks and the

B-A scale-free networks with connection densities of E=600 (**a**), *E*=1200 (**b**), *E*=1800 (**c**), *E*=2400 (**d**), *E*=3000 (**e**), *E*=3600 (**f**), *E*=4200 (**g**) and *E*=4800 (**h**).

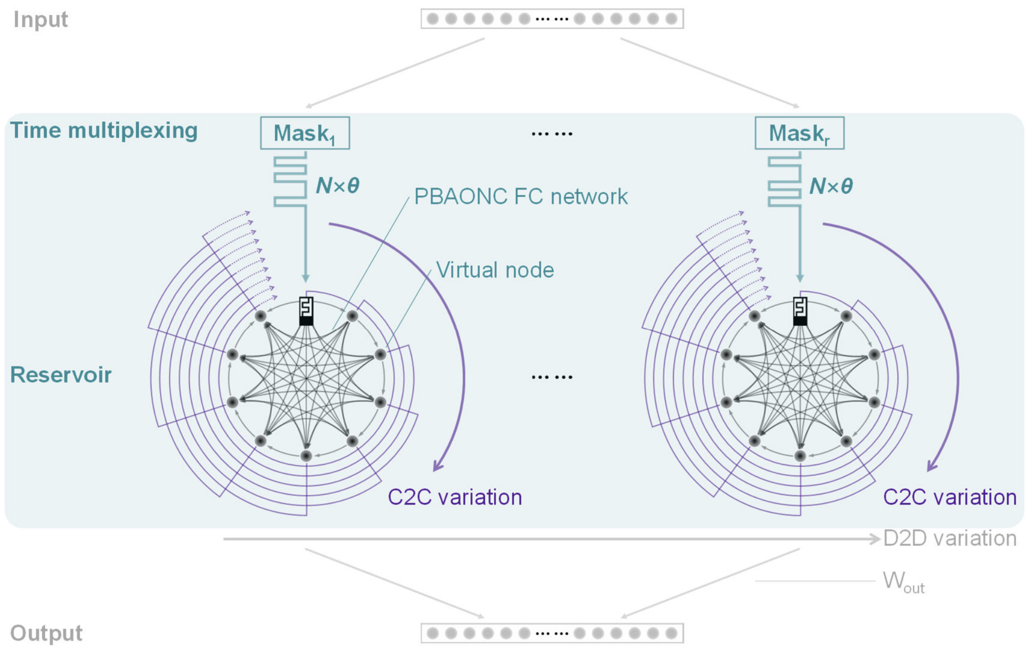

Fig. S9. PBAONC FC network reservoir. Schematic of the PBAONC FC network reservoir set for RC based on time multiplexing of the dynamic memristors.

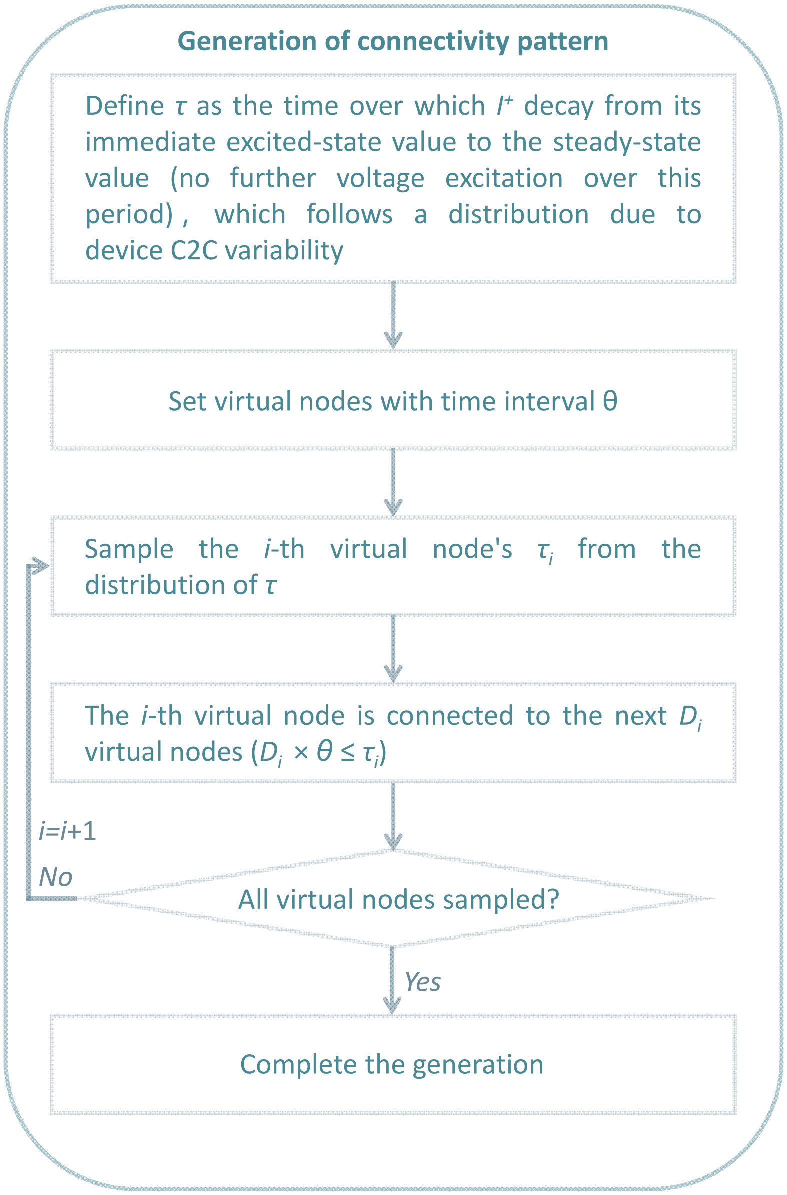

Fig. S10. Workflow of the generation of connectivity patterns.

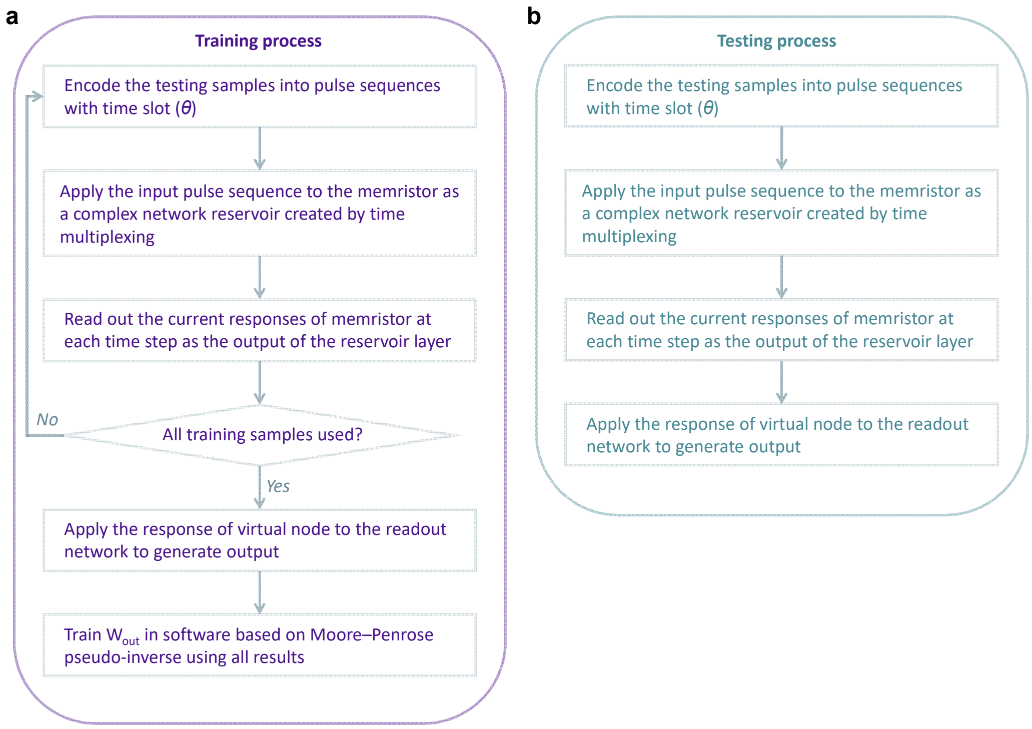

Fig. S11. Workflow charts of the training process and the testing process of reservoir computing based on the PBAONC complex network.

References

1. C. Du *et al.*, Reservoir computing using dynamic memristors for temporal information processing. *Nature communications* **8**, 1-10 (2017).
2. J. Moon *et al.*, Temporal data classification and forecasting using a memristor-based reservoir computing system. *Nature Electronics* **2**, 480-487 (2019).
3. Y. Zhong *et al.*, A memristor-based analogue reservoir computing system for real-time and power-efficient signal processing. *Nature Electronics* **5**, 672-681 (2022).
4. K. Liu *et al.*, An optoelectronic synapse based on α-In2Se3 with controllable temporal dynamics for multimode and multiscale reservoir computing. *Nature Electronics*, 1-13 (2022).
5. E. Bullmore, O. Sporns, The economy of brain network organization. *Nature reviews neuroscience* **13**, 336-349 (2012).
6. S. Boccaletti, V. Latora, Y. Moreno, M. Chavez, D.-U. Hwang, Complex networks: Structure and dynamics. *Physics reports* **424**, 175-308 (2006).
7. D. J. Watts, S. H. Strogatz, Collective dynamics of 'small-world'networks. *nature* **393**, 440-442 (1998).
8. D. S. Bassett, E. Bullmore, Small-world brain networks. *The neuroscientist* **12**, 512-523 (2006).
9. M. D. Humphries, K. Gurney, Network 'small-world-ness': a quantitative method for determining canonical network equivalence. *PloS one* **3**, e0002051 (2008).
10. P. Erdős, A. Rényi, On the evolution of random graphs. *Publ. Math. Inst. Hung. Acad. Sci* **5**, 17-60 (1960).
11. A.-L. Barabási, R. Albert, Emergence of scaling in random networks. *science* **286**, 509-512 (1999).
